# Cotton Terry Textiles with Photo- and Bio-Activity in a Model Study and Real Conditions

**DOI:** 10.3390/ma13153334

**Published:** 2020-07-27

**Authors:** Beata Gutarowska, Justyna Szulc, Edyta Matyjas-Zgondek, Piotr Kulpiński, Katarzyna Pielech-Przybylska, Anna Rygała, Anita Jachowicz, Eugeniusz Rutkowski

**Affiliations:** 1 Department of Environmental Biotechnology, Lodz University of Technology, Wólczańska 171/173, 90-924 Łódź, Poland; justyna.szulc@p.lodz.pl (J.S.); anna.rygala@p.lodz.pl (A.R.); anita.jachowicz@dokt.p.lodz.pl (A.J.); 2 Department of Mechanical Engineering, Informatics and Chemistry of Polymer Materials, Lodz University of Technology, Żeromskiego 116, 90-924 Łódź, Poland; edyta.matyjas-zgondek@p.lodz.pl (E.M.-Z.); piotr.kulpinski@p.lodz.pl (P.K.); 3 Institute of Technology Fermentation and Microbiology, Lodz University of Technology, Wólczańska 171/173, 90-924 Łódź, Poland; katarzyna.pielech-przybylska@p.lodz.pl; 4 Zwoltex Sp. Z O.O., Spacerowa 13, 98-220 Zduńska Wola, Poland; erutkowski@zwoltex.pl

**Keywords:** photocatalysis, self-cleaning materials, textiles, air quality, microorganisms, titanium dioxide, zinc oxide, volatile organic compounds

## Abstract

The aim of the study was to assess the photocatalytic (decompose staining particles, K/S values, the color differences, CIE L*a*b* color) and antimicrobial properties of textiles modified with TiO_2_ and ZnO nanoparticles (NPs) confirmed by X-ray diffraction, dynamic light scattering, SEM-EDX) in visible light conditions. The antimicrobial effectiveness of modified textiles under model conditions has been reported against 5 microorganisms: *Staphylococcus aureus, Escherichia coli*, *Bacillus subtilis*, *Candida albicans*, *Aspergillus niger* (AATCC Test Method 100-2004). In real conditions in bathrooms, significant biostatic activity was shown on the surface of the modified towels. The number of microorganisms decreased by 1–5 log to the level of 0–5 CFU/cm^2^ in the case of bacteria: *Enterobacteriaceae*, *Enterococcus*, the coli group and *E. coli*, *Pseudomonas*. Statistically significant reduction of the total number of bacteria and fungi (by 1 log), and the concentration of gases (NO_2_, CO_2_, CO) in the air of bathrooms was determined. The removal or reduction of volatile organic compounds (VOCs) concentration (SPME-GC-MS analysis) in the air above the modified towels has also been determined. It was found that the lighting type (natural, artificial), time (1.5 and 7 h/day), air humidity (RH = 36–67%) and light intensity (81–167 lux) are important for the efficiency of photocatalysis. Textile materials modified with TiO_2_ and ZnO NPs can be used as self-cleaning towels. They can also help purify air from microorganisms, VOCs and undesirable gases.

## 1. Introduction

Photocatalysis is defined as the chemical reactions under the action of ultraviolet, visible or infrared radiation in the presence of photocatalysts, which absorb light and are involved in chemical transformation (International Union of Pure and Applied Chemistry, 2004). The basic mechanism is the formation of ROS (reactive oxygen species) including radical reactive oxygen forms, such as superoxide anion radical (O_2_) and highly reactive hydroxyl radical (OH), and non-radical reactive oxygen forms, such as hydrogen peroxide (H_2_O_2_), singlet oxygen (^1^O_2_) and ozone (O_3_) [1,2].

Photocatalysis is considered as an efficient disinfection technique suitable for medical and pharmaceutical purposes, food protection, wastewater treatment, and the disinfection of water and indoor air [3,4,5]. TiO_2_ is the most popular photocatalytic material used as a self-cleaning material for surface coating in many applications, for example as an antimicrobial component of construction materials, glass, cement, polymers, ceramics, metals and paint coatings [6,7]. Textile materials with self-cleaning properties padded/coated with photocatalytic nano-compounds have received intensive scientific interest in recent decades [2,8]. Self-cleaning finishes have been applied to cotton [9,10,11], wool [12], PET [13] and mixed fibers [14].

Disinfection practices still deserve interest for the development of novel photocatalysts with the use of different experimental systems: TiO_2_ coating/doping with various metal nanoparticles (NP_S_), e.g., Zn, Cu, Pt, Ag, Cd, Mo and Co, and type of irradiation, e.g., visible light [15,16]. The most popular photocatalytic compound for textile applications among other semi-conductor metal oxides is TiO_2_, due to its high stability, high photocatalytic activity and non-toxicity [2,8]. Recently, an increase in the use of ZnO photocatalysts for textile finishing in the literature is observed [17]. Both TiO_2_ and ZnO photocatalytic NPs have a similar wide band gap that limits their activity in the UV region of light [18,19,20]. Metal/non-metal doping extends their spectral response to visible light [12,17,19,20,21]. Caschera et al. showed that europium doped TiO_2_ finished cotton textiles have photocatalytic activity in UV and visible light and were even more effective in visible light [22]. Zahid at al. demonstrated, that cotton fabric functionalized with manganese doped TiO_2_ showed excellent photocatalytic activity under visible light [23]. TiO_2_/diazonium/graphene oxide composite exhibited photocatalytic degradation of some dyes under visible light [24]. Moreover, the combination of ZnO and TiO_2_ metal oxides increases energy absorption, stability and their photocatalytic activities [12].

Photocatalysis proceeds via redox reactions generally by predominating oxidative reaction pathways. Besides the degradation of diverse organic and inorganic compounds, mainly dyes, toxic substances and volatile organic compounds (VOCs), an important aspect is microorganism inactivation by photocatalysis [16,25]. Recently, studies on photocatalytic activity have been intensively conducted on a wide spectrum of microorganisms, including many species of viruses (e.g., MS2 phage, RNA bacteriophage and phil164), bacteria (e.g., *Escherichia coli*, *Staphylococcus aureus*, *Bacillus subtilis*, total coliforms, *Salmonella* sp. and *Pseudomonas* sp.), fungi (e.g., *Fusarium* sp., *Candida albicans* and *Aspergillus niger*), and parasites (e.g., *Giardia intestinalis* and *Cryptosporidium parvum*) [5,6,16,26]. The main mechanism of photocatalytic antimicrobial reactions under light conditions is based on the complex chemical oxidation of the cell wall and cell membranes, mainly including reactions of ROS with major cell components, such as lipids and proteins [27,28].

It was found that the photocatalytic activity of TiO_2_ NPs onto textile materials is influenced by crystal structure [29], particle size and shape [18], TiO_2_ content [9], light type (UV and/or visible light [9], light intensity and irradiation time [10,11,30], as well as doping method [11]. The self-cleaning properties of textiles treated with ZnO NPs depend on ZnO concentration on the fibers and fabric preparations [31], relative humidity and water content on the fabric surface [17], as well as doping method [12].

In model studies, it was found that the efficiency of photocatalysis in the air is mainly influenced by parameters such as the type and surface of the photocatalytic material, the type, intensity and time of lighting, as well as the relative humidity of the air [3,7,32,33,34,35].

Thus far, research on the efficiency of photocatalysis of modified textile materials has focused mainly on model conditions, concerning water and air disinfection [4,16,34]. Studies on TiO_2_ modified photocatalytically active textiles concerned chitosan or cotton-chitosan and polyester, which showed biological activity against the following bacteria: *E. coli*, *S. aureus*, *B. cereus* [33,34].

Photocatalytic solutions of textile materials, especially towels in visible light have not been described in real conditions. Therefore, the purpose of the present study included: (1) the assessment of selected self-cleaning properties of modified textiles; (2) the assessment of the antimicrobial activity of photocatalytically (TiO_2_ and ZnO NPs) modified textile materials under model conditions; (3) the assessment of the microbiological contamination of the surface of photocatalytically modified towels, the microbiological contamination of air, and volatile organic compound and gas (carbon dioxide, carbon monoxide and nitrogen dioxide) concentrations in air under real conditions.

Modification of cotton terry textiles with above mentioned oxides is an attempt to obtain new kind of safe and environmentally friendly textile materials for multipurpose applications.

## 2. Materials and Methods

### 2.1. Textile Modification

A 100% cotton double-side terry woven fabric with three-weft assembled weave and declared surface mass of 500 g/m^2^ was made by ZWOLTEX Sp. z o.o., Zduńska Wola, Poland. The terry woven fabric was manufactured using basic warp and weft systems and the warp loop system. The basic warp and weft yarns were classic carded 100% raw cotton yarn, and open end 100% raw cotton yarn respectively. The finest warp yarn was 40/2 Nm, while the finest weft yarn was 25 Nm. The loop yarn was classic combed 100% raw cotton with the finest being 25 Nm.

#### 2.1.1. Fabric Pre-Treatment and Dyeing

Prior to modification, the fabric was pre-treated and reactive-dyed under industrial conditions using periodic finishing methods in rope form. First, the fabric was desized using the commercial desizing enzyme α-amylase—NewZyme SDL New (NewEnzymes, Pedrouço, Portugal). Next, scouring and peroxide bleaching were performed at reduced temperature in an alkaline bath maintaining the wetting and scouring agents stable in alkaline conditions (Cottoclarin OK, Pulcra Chemicals GmbH, Geretsried, Germany), using a peroxide decomposition stabiliser (Setalan Eco Bleach, Setas Kimyo Sanayi, Çerkezköy, Turkey), a sequestering-dispersing agent (Plexmet KDM, Achitex Minerva S.p.A, Vaiano Cremasco, Italy), hydrogen peroxide and sodium hydroxide. Subsequently, a bath peroxide residue clean-up, bio-finishing and reactive dyeing were performed. Bio-finishing was intended to enhance fabric appearance, improve softness and remove protruding fibers. A complex enzymes agent composed of neutral cellulase and catalase enzymes (NewCell Combi, NewEnzymes Pedrouço, Portugal) together with the sequestering-dispersing agent Plexmet KDM were used. The fabric was dyed using aminochlorotriazine reactive Procion HE-XL dyes (DyStar, Raunheim, Germany). The well-known hot brand reactive dye method was used. After dyeing, the fabric was neutralized using diluted acetic acid, rinsed, washed and rinsed several times again in order to remove unfixed reactive dyes from the fabric.

After the dyeing process, the terry woven fabric intended for making the control towels (without modification) was submitted to softening in an acidic bath containing cationic softeners Crosdurin GP (Eurodyte-CTC, Jodoigne, Belgium) and Persoftal Hydrosoft (Tanatex Chemicals B.V., Ede, The Netherlands). Afterwards, the fabric was dried at 150 °C for 5 min in open width form using the continuous tumbler dryer Penteck Energy X-Stream 3000/2800 (Penteck Textile Machinery Srl, Montemurlo, Italy).

#### 2.1.2. Fabric Modification Using Nanoparticles (NPs) with Photocatalytic Properties

Water dispersion of ZnO NPs (zincite crystal structure, NP mean size 86 nm and specific surface area 100 ± 10 m^2^/g) and TiO_2_ NPs (rutile crystal structure, N-doped crystal lattice, NP mean size 30 nm and specific surface area >60 m^2^/g) were purchased from Bochemie (Bohumín, Czech Republic) and Cinkarna (Celje, Slovenia) respectively. Dyed woven terry fabric was submitted to the functionalization process under industrial conditions using a continuous method. The fabric was open width padded using the Bianco M15/2019 Padder Model RW2800/2600 FWMM (Bianco S.P.A., Alba, India) in a bath containing 2.5 g/dm^3^ ZnO NPs, 2.5 g/dm^3^ N-doped TiO_2_ NPs, 40 g/dm^3^ Persoftal Hydrosoft (softening agent) and 0.6 g/dm^3^ Newalol PFN (wetting and deaerate agent). The pressure of squeezing shafts was 3 atm. Subsequently, the fabric was dried at 150 °C for 5 min in open width form using the continuous tumbler dryer Penteck Energy X-Stream 3000/2800 (Penteck Textile Machinery Srl, Alba, Italy).

**Figure 1:**
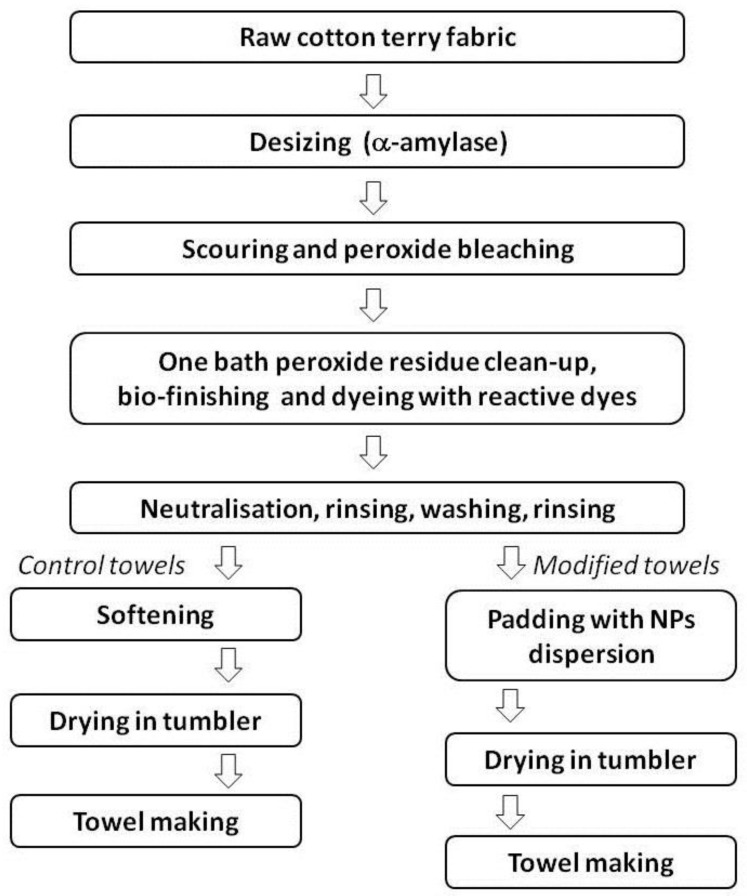
Schematic representation of finishing processes.

All finishing processes (pre-treatment, bleaching, dyeing, softening, modification) were carried out in ZWOLTEX Sp. z o.o., Zduńska Wola, Poland. A schematic representation of the finishing process is presented in Figure 1. The modification process with ZnO NPs and N-dopped TiO_2_ NPs of terry woven fabric is protected by Polish patent application No P.433366.

### 2.2. Material Characterisation

#### 2.2.1. Nanoparticles (NPs) Characterisation

X-ray diffraction (XRD) analysis was used to confirm the crystal structure of ZnO and TiO_2_ NPs in commercial dispersions. The diffraction patterns were collected using a PANalyticalX’Pert Pro MPD diffractometer in Bragg Brentano reflection geometry (Malvern Panalytical Ltd., Malvern, England). The diffractometer was equipped with Cu Kα radiation source (λ = 1.5418 Ĺ). Data was collected in the 2θ range of 5–90°.

Dynamic light scattering (DLS) was used to determine the size and the size distribution profile of ZnO and TiO_2_ particles in dispersions. The Particle Sizing System NICOMP 380, a product of Nicomp, (Santa Barbara, CA, USA) was used. The measurements were performed at the temperature of 23 °C. Data were acquired and processed by CW388 software (version 1.55, Nicomp, Santa Barbara, CA, USA).

#### 2.2.2. Textile Material Characterisation

Morphological observation of ZnO and TiO_2_ finished textiles was carried out using Scanning Electron Microscope (SEM) VEGA3-SBU (Tescan, Brno, Czech Republic). The elemental analysis of modified textiles was performed by energy dispersive X-ray spectroscopy (EDX) (Oxford Instruments Analytical, Abingdon, England). Before SEM-EDX investigation, the samples were placed on carbon plasters and coated with gold using Cressington Sputter Coater 108 auto (Cressington Scientific Instruments UK, Watford, England). In order to analyze the selected properties of the finished terry woven fabric towels, surface mass, time of water absorption and selected color fastness were determined. The surface mass was measured in accordance with [36]. Samples of terry fabric with dimensions of 10 cm × 10 cm were weighed and the results are the arithmetic mean of five weightings. The time of absorption was determined according to [37]. Samples of towels before and after modification with dimensions of 10 cm × 10 cm were laid on the surface of the water and the duration of the complete immersion of the samples was recorded. The time of absorption is the arithmetic mean of five measurements. Fastness to washing in 60 °C was carried out according to [38] was determined in accordance with [39]. A grey scale was selected to evaluate color fastness to washing and rubbing.

The ability of a cotton surface modified with photocatalytic compounds to decompose staining particles was tested. Self-cleaning properties were determined using 100% cotton woven fabric (made with bleached cotton yarn) of diagonal wave, desized and modified under laboratory conditions. A padding bath with the same composition as in industrial conditions was applied using a laboratory padding-squeezing machine (E. Benz, Zurich, Switzerland). The pressure of padding-squeezing rolls was 40 kG/cm. Then, the cotton samples were dried at 150 °C for 3 min using a laboratory stenter drying-curing machine, (E. Benz, Zurich, Switzerland). Prior to the self-cleaning experiment, modified cotton samples were stored in the dark for 2 days.

Blackcurrant nectar containing 25% blackcurrant juice (Hortex Sp. z o.o., Warszawa, Poland) was selected to assess self-cleaning properties. Juice staining was applied onto the fabric surface using stamp printing. Printed samples were dried in the dark. One-half of stained samples (control and modified) were irradiated for 6 h using a 160 W mercury lamp (Solar Glo PT2193, Exo Terra, Montreal, QC, Canada) with a spectrum similar to the spectrum of sunlight at a distance of about 30 cm above the sample surface. The UVA/B radiation was at an intensity of approximately 1.4 mW/cm^2^. The UVA/B radiation intensity was monitored by means of a UV-AB ST513 SENTRY radiation meter (Sentry Optronics Corp., Taipei, Taiwan). The irradiated samples were sprayed with distilled water before and after each hour of irradiation. The other half of stained samples were stored in the dark. The stained samples (irradiated and unirradiated) were subjected to color measurements using the Spectraflash 300 colorimeter (DataColor, Rotkreuz, Switzerland). Prior to measurement, the instrument was calibrated according to the manufacturer’s instructions.

The light reflectance R(%) was measured in the range of 400–700 nm. The self-cleaning action was monitored by comparing the K/S values, the color differences and CIE L*a*b* color system between irradiated and unirradiated stained samples. L* is a coordinate of lightness, a* and b* are termed opponents to color axes.

K/S values were determined using Equation (1):

K/S = (100 − R)^2^/(200R) (1)

where: R—light reflectance

The color differences were determined using Equation (2):

ΔE* = [(ΔL*)^2^ + (Δa*)^2^ + (Δb*)^2^]^1/2^ (2)

where:

ΔL*—lightness differences between reference and measured samples

Δa*—red/green differences between reference and measured samples

Δb*—yellow/blue differences between reference and measured samples.

### 2.3. Microorganisms

In the model experiment, five microorganisms from the American Type Culture Collection (ATCC) and the National Collection of Agricultural and Industrial Microorganisms (NCAIM): *Staphylococcus aureus* ATCC 6538, *Escherichia coli* ATCC 10,536, *Candida albicans* ATCC 10231, *Aspergillus niger* ATCC 16,404 and *Bacillus subtilis* NCAIM 01,644 were used. The selected microorganisms represent different morphology, physiology and sensitivity to disinfection.

### 2.4. Characteristics of Real Conditions

The impact of control and modified towels on the amount of microorganisms (bacteria, fungi) in the air, and the concentration of selected gases (carbon dioxide, carbon oxide, nitrogen dioxide, ozone) in the air during seven days of use was assessed in two different bathrooms: Bathroom No. 1 (cubature—12.86 m^3^, artificial light, central heating, mechanical ventilation, number of users—4 adults); Bathroom No. 2 (cubature—40.5 m^3^, natural light—1 window 0.9 × 0.9 m, central, gas and floor heating, mechanical ventilation, number of users—2 adults, 2 children). Two towels were used in each bathroom (control towels during seven days, then modified towels for the next seven days). Towels were hung the whole surface on bathroom hangers. Before each series of experiments (with control end modified towels), bathrooms were cleaned the same way with the same cleaners. In the bathrooms, measurements of air temperature, relative humidity of the air (RH), light intensity and lighting time were made every day (about 10 cm above the surface of the towel). Air humidity and temperature were measured using an HL-1D hygrometer (Rotronic, Bassersdorf, Switzerland) and light intensity using a BL-10L luxometer Voltcraft BL-10L, (Conrad Electronic, Kraków, Poland). In addition, concentrations of selected gases in the indoor air (carbon dioxide, carbon oxide, nitrogen dioxide, ozone) were measured daily using a gas analyser X-am 5600 (Dräger, Lübeck, Germany). The concentration of ozone in the air in the bathrooms was at a minimal level within the range of 0.00–0.04 ± 0.02 ppm. The microclimatic and lighting parameters (average values from three daily measurements) during the experiment in the bathrooms are given in Table 4.

### 2.5. Antimicrobial Activity of Textiles—Model Study

The antimicrobial activity of textiles under model conditions was determined by the AATCC Test Method 100-2004 [40] according to previous studies [41]. The densities of inoculums were established by the culture method. The densities for the tested microorganisms were: 5.7 × 10^7^ CFU/mL (*B. subtilis*), 7.3 × 10^7^ CFU/mL (*A. niger*), 2.3 × 10^8^ CFU/mL (*C. albicans*), 7.2 × 10^8^ CFU/mL (*S. aureus*), 4.4 × 10^9^ CFU/mL (*E. coli*). The suspension of microorganisms (0.1 mL) was inoculated on the surface of the modified and control samples (area 4 cm^2^). Next, the samples were incubated at temp. 30 ± 2 °C and RH: 80 ± 1% without lighting (in a Binder-720 climate chamber, Tuttlingen, Germany). An additional sample with *E. coli* was incubated in room conditions (RH = 43 ± 5%, temp = 21 ± 2 °C, light intensity 69.5 ± 10 lux, lighting time 420 min). Samples were collected after time *t* = 0 h and 24 h, transferred into saline (50 mL sterile 0.85%) and shaken (5 min). Then, the method of serial dilutions in saline (from 10^−2^ to 10^−6^) and the culture method were performed. The microorganisms were cultured in the following conditions: MEA medium (Malt Extract Agar, Merck, Darmstadt, Germany), temp. 27 ± 2 °C, 48–72 h (number of fungi); TSA medium (Tryptic Soy Agar, Merck, Darmstadt, Germany), temp. 30 ± 2 °C, 24–48 h (number of bacteria). After incubation, the colonies were counted and the result was given as colony forming units per 4 cm^2^ of sample (CFU/sample). The tests were performed in three independent replicates for each variant. Survival index (N) after 24 h incubation and antimicrobial biostatic activity (A) were determined.

The microbial survival index was determined using Equation (3):

N = (N_t_/N_0_) × 100% (3)

where:

N_0_—number of microorganisms on textile material (CFU/sample) at time *t* = 0 h

N_t_—number of microorganisms on textile material (CFU/sample) at time *t* = 24 h

Biostatic activity was determined using Equation (4):

A = log N_C_/N_m_ (4)

where: N_C_—number of microorganisms on textile material (CFU/sample) after incubation on the control textile material; N_m_—number of microorganisms on textile material (CFU/sample) after incubation on modified textile material.

The results of biostatic activity were compared to criteria using normative findings in order to determine the biostatic effect of disinfectants against microorganisms [42]. Activity below 1 was considered low, activity at the level of 1–3 was considered medium, and above 3 it was high, assuming that the value of 1 means a decrease in the number of microorganisms by one logarithmic scale.

### 2.6. Microbial Analysis of Towel Surface—Real Conditions

The assessment of microbiological contamination of the control and modified with photocatalytic compounds towels was carried out after seven days of use by three people (each person used a control towel for seven days, and then a modified towel for the next seven days). Towels were used in bathrooms No. 1–2. Then, 200 cm^2^ of towel samples were taken from each towel for testing. They were shaken in saline, diluted and cultured, and incubated in various conditions, which allowed the assessment of their microbiological contamination. The following media and incubation conditions were used: MEA (Malt Extract Agar, Merck, Darmstadt, Germany) with chloramphenicol (temp. 27 °C, 48–72 h (total number of fungi); TSA (Tryptic Soy Agar, Merck, Darmstadt, Germany) with nystatin, temp. 37 °C, 24–48 h (total number of bacteria); TBX Agar (Tryptone Bile X-Glucoronide, Oxoid, Basingstoke, UK) and Chromocult Coliform Agar (Merck, Darmstadt Germany), temp. 37 °C, 24–48 h (coli group and *Escherichia coli*); VRBD (Crystal-Violet Neutral-Red Bile Dextrose Agar, Merck, Darmstadt, Germany), temp. 37 °C, 24–48 h (*Enterobacteriaceae*); Cetrimide Agar (Merck, Darmstadt, Germany), temp. 37 °C, 24–48 h (*Pseudomonas*); Slanetz and Bartley Agar (Merck, Darmstadt, Germany), temp. 37 °C, 24–48 h (*Enterococcus*). After incubation, the colonies were counted and the result was given in CFU/cm^2^. The results are the arithmetic mean of three towels and three replicates for each determination.

Bacteria and fungi constituted the dominant microflora on the tested towels and were selected for identification (frequency of occurrence—above 30% of all isolates). Bacterial identification was made on the basis of observation of macroscopic features, Gram staining, catalase and API STAPH tests (bioMérieux, Marcy l’Etoile, France). Yeasts were identified using the API C AUX test (bioMérieux, Marcy l’Etoile, France). Identity compliance % ID was 97.4–99.9% for bacteria and 78.4–98.3% for yeasts.

### 2.7. Microbial Air Contamination Assessment—Real Conditions

The number of bacteria and fungi in the air during seven days of use of the control and modified towels in two bathrooms were determined. Samples of the air (volume 100 L) after 1, 4 and 7 days of use were taken using a Mass 100 Air Sampler (Merck, Germany). Microorganisms were cultured in the following conditions: MEA with chloramphenicol, temp. 27 ± 2 °C, 48–72 h (total number of fungi); TSA with nystatin, temp. 30 ± 2 °C, 24–48 h (total number of bacteria). After incubation, the colonies were counted and the result was given in CFU/m^3^. The tests were performed in three replicates at a distance of 50 cm from the towels.

### 2.8. Analysis of Volatile Organic Compounds—SPME-GC-MS

The volatile compounds in the towels (after seven days of use) were collected via solid phase microextraction (SPME) and then analysed with gas chromatography coupled with mass spectrometry (GC-MS). Prior to extraction, a towel sample was cut (1 g), placed in a glass vial (22 mL) and closed tightly. The sample was equilibrated for 15 min at 40 °C and then volatiles were collected onto an SPME fiber (DVB/CAR/PDMS, 50/30 μm, 1 cm length; Supelco, Bellefonte, PA, USA) for 15 min, at the same temperature. Prior to each extraction, the fiber was conditioned for 10 min, at 250 °C. After extraction, the fiber was placed into a GC injection port for volatile desorption (5 min, 250 °C). The sample injection was performed in splitless mode. Gas chromatography analysis was carried out using Agilent 7890A (Agilent Technologies, Santa Clara, CA, USA). Volatile compound detection was carried out using mass spectrometer Agilent MSD 5975C (Agilent Technologies, Santa Clara, CA, USA). The DB-1 ms capillary column (30 m × 0.25 mm × 0.25 µm, Agilent Technologies Santa Clara, CA, USA) was used for volatile compound separation. The oven temperature program was as follows: 30 °C for 6 min, increasing to 240 °C at a rate of 10 °C/min and held for 2 min. The temperature of the mass spectrometer ion source, transfer line and quadrupole analyzer was set at 230, 250 and 150 °C respectively. The electron impact energy was 70 eV. The mass spectrometer operated in the full scan mode. Qualification of volatile compounds was performed by comparison of the obtained spectra with reference mass spectra from the NIST/EPA/NIH Mass spectra library (2012; version 2.0 g.) and the Kovàts retention indices (RI) calculated for each peak with reference to the n-alkane standards (C6–C20). Each towel sample was analysed in triplicate.

## 3. Results

**Figure 2:**
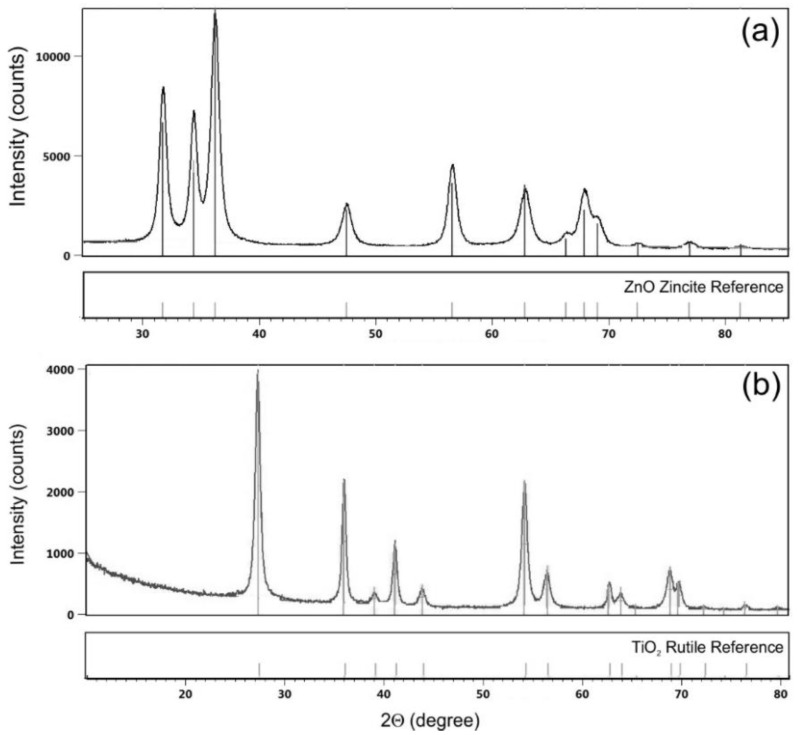
XRD patterns of (**a**) ZnO and (**b**) TiO_2_ NPs commercial dispersion.

The results of XRD measurements of ZnO and TiO_2_ commercial dispersions of NPs are shown in Figure 2. The XRD pattern of ZnO NPs (Figure 2a) shows diffraction peaks at 2Θ = ca. 31°, 34°, 36°, 47°, 56°, 62°, 66°, 67°, 69°, 72°, 77° and 81°, which are characteristic for hexagonal zincite ZnO [43]. The XRD pattern of TiO_2_ NPs (Figure 2b) shows diffraction peaks at 2Θ = ca. 27°, 36°, 39°, 41°, 44°, 54°, 56°, 63°, 64° and 69°. The XRD pattern are characteristic for tetragonal rutile TiO_2_ [44,45].

The results of size and particle size distribution of ZnO and TiO_2_ NPs in commercial dispersions obtained by the DLS measurements are shown in Table 1.

**Figure 3:**
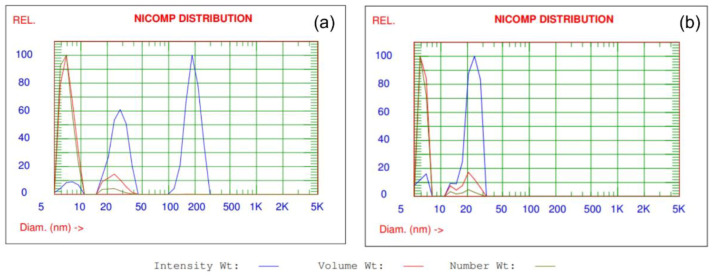
Nicomp distribution analysis of the intensity-, volume- and number-weighted data collected for (**a**) ZnO and (**b**) TiO_2_ NPs in commercial dispersions.

The Nicomp distributions of the intensity-, volume- and number-weighted data collected for ZnO and TiO_2_ NPs in dispersions are shown in Figure 3. The assumptions of DLS Nicomp analysis were described in details by [41]. DLS analysis of particles size and size distribution confirms that ZnO and TiO_2_ are in dispersions in the form of nanoparticles. The size of ZnO NPs and TiO_2_ NPs in dispersions are in the range of 7.1–23.8 nm and 6.3–21.4 nm, respectively. The biggest percentage fine fraction of particles, both for ZnO and TiO_2_ NPs are observed in dispersions in the range of 85.4–87.2% for volume weight intensity and 95.3–96% for number weight intensity.

**Figure 4:**
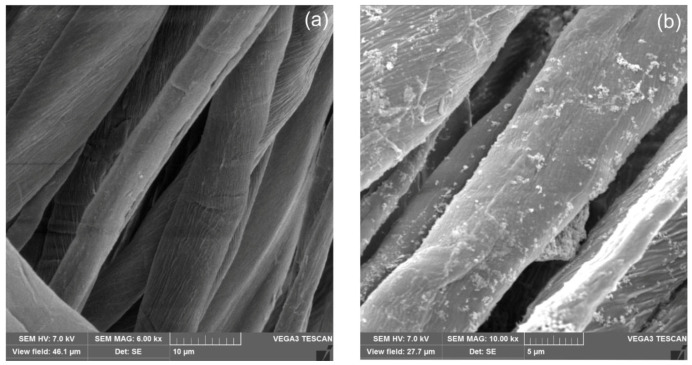
SEM images of (**a**) unmodified and (**b**) ZnO and TiO_2_ modified cotton terry towels.

The morphology of ZnO and TiO_2_ modified cotton terry towels was examined with the scanning electron microscopy (SEM) technique. Figure 4 shows SEM images of unmodified and modified cotton terry towels with magnification of ×6000 and ×10,000, respectively. It can be seen the nanoparticles homogeneously (Figure 4b) dispersed on the fibers surface. Unfortunately, a magnification of SEM microscope did not allow to clearly shown the nanoparticles as were stated by the DLS analysis.

**Figure 5:**
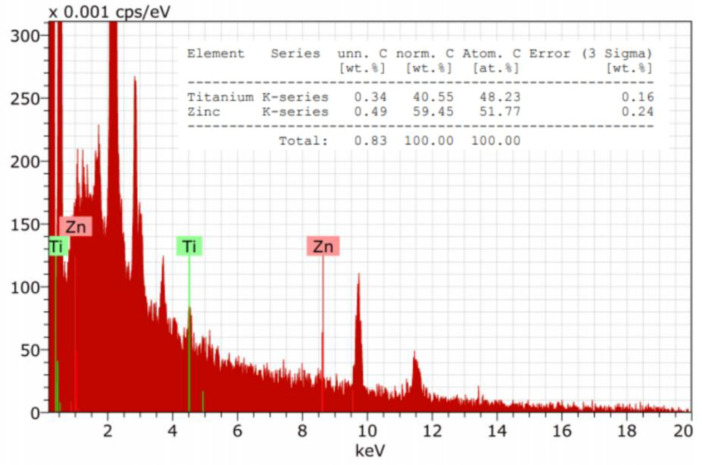
Example of EDX spectra of cotton terry towels modified with ZnO and TiO_2_ NPs.

The EDX studies (Figure 5) confirmed the presence both Zn and Ti elements on the surface of modified cotton terry towels. 

The evaluated 100% cotton towels were characterized by a high level of color fastness to washing in 60 °C and dry and wet rubbing (Table 2). No influence of the modification process with ZnO/N-doped TiO_2_ NPs on the level of color fastness was observed. The results showed that modified towels had shorter adsorption time and higher surface mass than unmodified ones. The tested towels met the standard’s requirements for the level of color fastness to washing, dry and wet rubbing and absorption time [46].

The results on the self-cleaning properties of modified materials assessed on diagonal cotton wave fabric using blackcurrant juice are shown in Table 3. The color variation of stained samples was assessed using K/S values, CIE L*a*b* coordinates and the color difference ΔE* before and after 6 h of UV/VIS irradiation. The L* value refers to sample lightness from 0 (black) to 100 (white), whereas a* and b* values characterize color variation: from green (−a*) to red (+a*) and blue (−b*) to yellow (+b*) respectively. Color difference ΔE* is a numerical comparison between the reference color and that of the tested samples. According to the well-known Kubelka–Munk theory, K/S value is linearly related to colorant concentration in the fabric.

Almost 60% reduction in K/S value after 6 h UV/VIS irradiation was observed for modified fabrics in comparison to 26% reduction for untreated fabric. Increase in L* values for control and modified blackcurrant juice stained fabrics indicated that both samples became lighter after UV/VIS irradiation. The L* value ranged from 73.38 and 73.57 to 75.44 and 80.70 for untreated and modified samples respectively. The modified sample, however, was lighter after irradiation than the untreated sample. ΔE* indicated significant color differences after irradiation (the higher the ΔE* value, the greater the color difference). UV/VIS irradiation significantly affected shade changes in the modified sample in comparison to the control, which was characterized by small shade changes. The b* movement from −1.86 to 6.26 and the a* movement from 9.56 to 2.52 represent a shift from red towards yellow after irradiation due to photodegradation of the color compound. This confirmed that the self-cleaning action of the fabric surface modified with ZnO/TiO_2_ NPs has photocatalytic properties.

**Figure 6:**
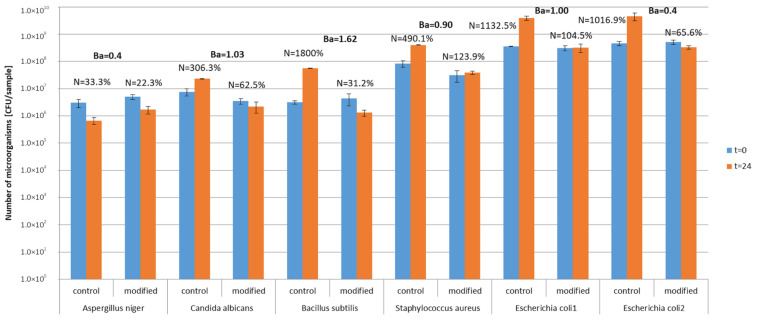
Antimicrobial activity of textiles—study in model conditions. N—survival index; Ba—biostatic activity; 1 sample incubated without lighting, 2 sample incubated with natural lighting.

Study in model conditions showed that microorganisms survived on textiles in both control and modified materials (Figure 6). Survival indexes on control textiles ranged from N = 33.3% (*A. niger*) to N = 1800% (*B. subtilis*), which indicates that the number of microorganisms increased after 24 h incubation on textile materials. On photocatalytically modified textile materials, the survival index was N = 22.3–123.9% and the number of microorganisms decreased by one logarithmic scale after 24 h of incubation. Biostatic activity of photocatalytically modified textiles against the tested microorganisms in model conditions was on the low and medium level A = 0.4–1.62. The lowest activity of modified materials was determined for mold *A. niger* and the highest for bacteria *B. subilis*. Comparing two samples of the modified textile materials with *E. coli* under different lighting conditions (without light and under natural light), it was found that the biostatic activity of the material against this bacterium increased from A = 0.9 in conditions without light to A = 1 in natural light conditions after 24 h of incubation.

Study in real conditions showed a much higher effect of photocatalytic activity against microorganisms than the model study. The microclimatic and lighting conditions in Bathroom No. 1 with artificial lighting and Bathroom No. 2 with natural lighting were different (Table 4): Bathroom No. 1, average RH = 65.6–66.8%, temp. 21 °C, light intensity 152.8–166.7 lux, lighting time 80.7–87.8 min/day; Bathroom No. 2, average RH = 36–44.5%, temp. 23 °C, light intensity 92.3–95.0 lux, lighting time 420 min/day. Microclimatic and lighting conditions in the bathrooms during the experiments with control and modified towels were the same (Table 4).

**Figure 7:**
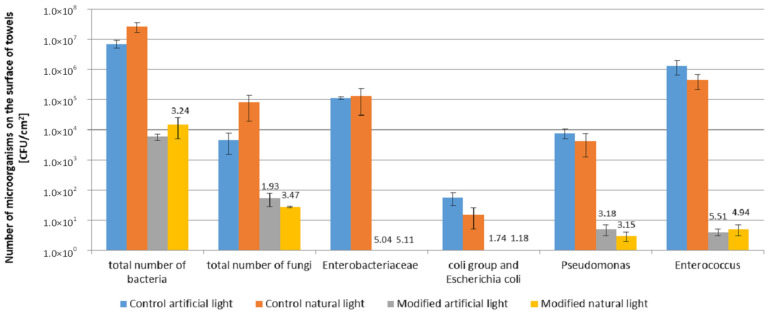
Microbial contamination of control and modified towels after seven days use—real conditions. The numbers on the figure are calculated biostatic activity.

The microbial contamination of control towels used under real conditions in the bathrooms described was higher compared to the photocatalytically modified towels (Figure 7).

Moreover, a significantly higher antimicrobial effect of modified towels was found under real conditions than in model conditions. The number of microorganisms on control towels after seven days of use was: total number of bacteria (7.0 × 10^6^–2.6 × 10^7^ CFU/cm^2^), total number of fungi (4.5 × 10^3^–8.0 × 10^4^ CFU/cm^2^). The dominating bacteria were: *Enterobacteriaceae* (1.1 × 10^5^–1.3 × 10^5^ CFU/cm^2^), *Enterococcus* genus (4.4 × 10^5^–1.3 × 10^5^ CFU/cm^2^) and *Pseudomonas* genus (4.2 × 10^3^–7.5 × 10^3^ CFU/cm^2^). The least number of the coli group and *Escherichia coli* on control towels after 7 days (1.5 × 10^1^–5.5 × 10^1^ CFU/cm^2^) was determined. The high number of bacteria on control towels is worth noting, namely above 10^5^ CFU/cm^2^ of the *Enterobacteriaceae*, *Enterococcus* genus. No significant differences were found between the microbial contamination of control towels used in both bathrooms (Figure 7). In the case of photocatalytically modified towels after seven days of use, the number of all groups of microorganisms was lower than in the case of control towels. The reduction of the microorganism number on modified towels compared to the controls was 3–6 log, which indicates high biostatic activity, much higher than in the model study (1 log). The biostatic activity of modified towels used in real conditions was higher against all groups of microorganisms tested (A = 1.18–5.51) than textile materials in the model study (A = 0.4–1.62).

The highest reduction of the number of microorganisms on modified towels was determined for bacteria. The number of *Enterobacteriaceae*, *Enterococcus*, the coli group and *E. coli*, and *Pseudomonas* decreased to 0–5 CFU/cm^2^. A low number of fungi on the modified towels was also noted (2.7 × 10^1^–5.3 × 10^1^ CFU/cm^2^), while the number of bacteria was on the level of 5.7 × 10^3^–1.5 × 10^4^ CFU/cm^2^.

There were no differences in microbiological contamination on the surface of modified and control towels in both types of bathrooms with artificial and natural lighting.

The identification of dominant microorganisms on modified towels after seven days of use (frequency above 30%) showed the presence of *Staphylococcus* (*S. hominis*, *S. xylosus, S. saprophyticus, S. lentus, S. warneri*) and *Kocuria varians* bacteria, while *Candida parapsilosis* and *Cryptococcus humicola* were the dominant fungi. Microorganisms isolated from the modified towels belong to the natural skin microflora.

Microbiological air contamination and the concentration of gases in the air of bathrooms during the use of the towels varied depending on the type of towels and the type of lighting in the bathroom (Table 5). In bathrooms where control towels were used, the total number of bacteria was 1.47 × 10^3^–2.05 × 10^3^ CFU/m^3^ and the total number of fungi 6.4 × 10^1^–1.45 × 10^2^ CFU/m^3^. These levels of microbial air contamination remained constant during the experiment. The number of bacteria in the air of both bathrooms and the number of fungi in the air of the bathroom with artificial lighting were higher than the level of 10^3^ CFU/m^3^ for bacteria and 10^2^ CFU/m^3^ for fungi, which indicates a high level of microbial air contamination. When modified towels were used, a decrease in the number of microorganisms in the air of bathrooms during the experiment was found, and the average number of microorganisms was: for bacteria 3.46 × 10^2^–6.97 × 10^2^ CFU/m^3^ and for fungi 1.40 × 10^1^–3.55 × 10^1^ CFU/m^3^. A statistically significant decrease in the number of bacteria and fungi in the air was found by almost 1 log compared to the bathroom where control towels were used. Moreover, significantly fewer microorganisms were found in the air in the bathroom with natural lighting where modified towels were used, than in the bathroom with artificial lighting, probably due to the greater activity of photocatalytic processes in natural lighting conditions. This is because the duration of lighting was much longer than in the bathroom with artificial lighting (Table 5).

The gas concentration in the air of bathrooms where control towels were used (NO_2_: 0.02–0.44 ppm; CO_2_: 0.12–0.16 vol%; CO: 22.22–35.44 ppm) was statistically higher than in the air of bathrooms where modified towels were used (NO_2_: 0.00–0.11 ppm; CO_2_: 0.09–0.12 vol%; CO: 8.33–23.44 ppm) (Table 5). The lowest concentrations of gases in the air were found in the bathroom with natural lighting, where photocatalytically modified towels were used.

One-hundred-and-eleven volatile organic compounds were detected in the air above the tested towels. Most could be of cosmetic origin (96 compounds), but some were of textile (38 compounds) and microbial (29 compounds) origins (Table 6). Among the identified volatile compounds, 25 were detected in all samples and 36 in towel samples before use (textile origin). Among all the identified compounds, the concentration of 66 volatile compounds in the air above the modified towels decreased compared to the control towels. An increase in the concentration of VOCs in the air was found for 32 compounds, while no change was found for 13 compounds. Moreover, 17 compounds were not detected in the air above modified towels compared to the control towels. The results indicate that 15% of all VOCs were removed from the air over the modified towels, and that the concentration of 58% of all compounds was lower in the air as a consequence of the photocatalytic activity of the modified towels.

## 4. Discussion

The objective of this study was to evaluate the photocatalytic and biological activity of 100% cotton terry towels modified with ZnO and N-doped TiO_2_ NPs in model and real conditions. All stages of terry fabric finishing processes and towel making were carried out in industrial conditions according to patented finishing technology. Cotton terry towels were surface modified using commercial dispersions of ZnO and TiO_2_ NPs. ZnO NPs present in dispersion is in the zincite crystal structure, while TiO_2_ NPs is in rutile crystal structure as was stated from XRD analysis. The XRD measurements confirmed the declared by manufactures crystal phases of semiconductors in both commercial dispersions. The differences in size and particles size distribution of ZnO and TiO_2_ in commercial dispersions declared by the manufacturers and obtained from the DLS analysis may be related to the different measurement techniques used.

The analysis of SEM micrographs of modified fabric has shown that the described padding- squeezing method of terry fabric modification allows the obtainment of NPs randomly distributed on the fibers surface, mainly in nanoscale size with a contribution of submicro-particles. Energy dispersive X-ray spectroscopy (EDX) was used to identify the elements present on the fabric surface. The determined amount of Zn and Ti atoms on the surface of the modified cotton terry towels corresponds well with the composition of the finishing bath, as was confirmed by EDX analysis.

It was observed that the modification process affects the fabric surface mass and time of absorption. Increases in surface mass and decreases in absorption time are probably connected with the deposition of NPs into fiber surfaces.

The photodegradation of blackcurrant juice staining on the surface of diagonally woven fabric modified with ZnO and N-dopped TiO_2_ NPs under UV/VIS irradiation was confirmed. Most semi-conductors, such as ZnO and TiO_2_, are activated by light only in the UV region, which limits their use in textiles for outdoor application. For indoor applications, where artificial light is predominantly in use, only visible light responsive photocatalysts can be used. For instance, N-doping of TiO_2_ NPs extends the spectral response of photocatalysts to visible light [12,19,20]. ZnO and TiO_2_ deposited in this study onto fiber surfaces show synergic photocatalytic effects. The authors are aware that this type of modification can cause the random distribution of both types of semiconductors on the fibers surface. However, preliminary research results indicate the synergic activity of both semiconductors, but this topic is beyond the scope of this publication.

In model conditions of the photocatalytically modified textiles, low and medium biostatic activity (A = 0.4–1.62) was observed towards the five tested microorganisms. Their sensitivity was ranked in the following order: *B. subtilis*, *S. aureus*, *E. coli*, *C. albicans* and *A. niger*. Hence, higher sensitivity was demonstrated for Gram-positive bacteria than for Gram-negative ones, which complies with the studies by Pal et al. [47] and Seven et al. [48]. Other authors state that Gram-negative bacteria are more sensitive to photocatalysis than Gram-positive ones, which results from the much thinner structure of the cell membrane (fewer layers of peptidoglycan) in Gram-negative bacteria [4,6,49]. In the majority of papers, as confirmed by the present model studies, yeast and mold are indicated as organisms that are less sensitive to photocatalysis than bacteria, which can be attributed to the different structure of the cell envelopes and the formation of resistant spores by mold [7].

The discrepancies between the literature and the present data concerning bacterial sensitivity to photocatalysis may result from different model conditions (different photocatalysts, lighting and microclimatic conditions used in the tests). Consequently, various processes can take place, whose mechanisms are responsible for the microorganism growth inhibition effect. In the present model studies, the direct impact of biocides in the form of TiO_2_ and ZnO on the microorganisms was the main biological mechanism on the material surface. The present model studies were carried out under high relative humidity conditions (RH = 80%), with no access to light (incubation in a climate chamber), which determines the biological effect by metal ion penetration into the microorganism cells, multi-dimensional action through binding Ti^2+^ and Zn^+^ cations to proteins and DNA, inhibition of protein biosynthesis and, consequently, inhibition of metabolism. The mechanism of such activity upon direct contact of the microorganisms with Ag, Zn and Ti metal nanoparticles has been described in the literature [50,51].

The model studies in this paper concerning *E. coli* bacteria incubated on the modified textiles in conditions of visible light and RH = 43% confirmed a minor increase in biostatic activity (A = 1), compared to conditions of darkness and high humidity RH = 80% (A = 0.9), which may support the direct biocide impact effect through photocatalytic processes induced by visible light.

The antimicrobial activity in the model conditions was low or medium. It can be considered only as biostatic effect, no biocidal effect was obtained, which requires reduction of 2 log reduction. Microorganisms survived on textiles in model conditions much better than in real conditions. The survival of microorganisms on bioactive textiles in model conditions depends on many factors, one of them is strain sensitivity, the presence of additional nutrients in the form of residues laboratory medium, optimal growth conditions (temperature, humidity) that have a protective effect on microorganisms compared to real conditions (environmental strains, lack of nutrients, various climatic conditions).

Currently, emphasis is placed on new photocatalytic solutions, e.g., using two kinds of photocatalysts as nanoparticles that will be photocatalytically active in visible light [52,53,54]. Thus, in the present studies, the tested textile fabric was modified with TiO_2_ and ZnO nanoparticles. Light activates photocatalytic processes, contrary to the direct biocide action mechanism. The photocatalysis mechanism is mainly based on the formation of ROS, which draws out the hydrogen atoms from C–H bonds or attacks the unsaturated chemical bond [55]. ROS oxidizes proteins and lipids in cell envelopes and leads to changes in the cell surface and the release of ions from microorganism cells [56,57]. Another mechanism, which can be responsible for cell death, is oxidation of the intracellular coenzyme A (CoA), which inhibits cell respiration [50] and attacks intracellular components directly causing alteration of the protein structure [58].

To date, there is no mention in the literature of textiles with photocatalytic properties in visible light under real conditions. For this reason, this paper focuses on testing an industrial solution (Polish Patent Application No P.433366) in cooperation with a towel manufacturing company. The tests were conducted in two types of bathrooms used by a similar number of people. The lighting and air humidity in the bathrooms were different. In the bathroom with artificial lighting the conditions were as follows: average air humidity RH = 65.6–66.8%, temp. 21 °C, light intensity 152.8–166.7 lux and lighting time 80.7–87.8 min/day. In the bathroom with natural lighting the following conditions were met: average RH = 36–44.5%, temp. 23 °C, light intensity 92.3–95.0 lux and lighting time 420 min/day.

On the surface of the modified towels after seven days of use, a high reduction in the number of microorganisms was observed, especially for *Enterobacteriaceae, Enterococcus,* coli group and *E. coli* and *Pseudomonas.* The number of microorganisms decreased from 10^1^–10^6^ CFU/cm^2^ to 0–5 CFU/cm^2^, which denotes their almost complete removal. Biological activity towards these groups of microorganisms has also been demonstrated in the literature in the model studies of different photocatalytic systems [16,32]. The identification of microorganisms isolated from the surface of the towels modified with TiO_2_ and ZnO after seven days of use revealed that *Staphylococcus* (*S. hominis*, *S. xylosus*, *S. saprophyticus*, *S. lentus*, *S. warneri*) and *Kocuria varians* were the dominating bacteria. These occur naturally in the skin microflora (*Staphylococcus*) and in the air (*Kocuria*), and can be less sensitive to the direct impact of biocides and photocatalytic processes than *Enterobacteriaceae*, *Enterococcus*, coli group and *E. coli* and *Pseudomonas.* In the present study, under real conditions, a statistically significant decrease in the number of fungi from 10^3^ CFU/cm^2^ to 10^1^ CFU/cm^2^ was observed on the towel surfaces. The identification of the dominant fungi revealed the presence of *Candida parapsilosis* and *Cryptococcus humicola* yeast originating from human skin. The literature indicates that yeast microorganisms are moderately sensitive to photocatalysis [32]. No mold was detected on the towel surfaces. The present study conducted under real conditions of towel use reveals the self-cleaning effectiveness of photocatalytically modified towels and the sensitivity of the selected groups of microorganisms (Gram-negative bacteria in particular), which reside on these surfaces. One should bear in mind that the photocatalytic self-cleaning of towels can be affected by the number, types and condition of physiological microorganisms, environmental pH and the presence of organic contaminants.

In real-condition studies, we can assume that there are two impact mechanisms on microorganisms: (*i*) direct reaction of biocides on the towel surface, and (*ii*) light-induced catalysis in the air in the bathrooms where the modified towels were used. Lack of statistically significant differences in the biostatic activity of the towels used in both bathrooms can suggest that the main mechanism, which occurred on the material surfaces, involved the direct impact of TiO_2_ and ZnO nanoparticles on microorganism cells.

In the present study, it was discovered that microclimate parameters and lighting in the rooms have a significant impact on photocatalytic processes in the air. Similar relations were described in the model studies using different photocatalytic arrays, which were significantly affected by air humidity, and lighting type and time [30,31]. In the present real-condition studies, the duration of exposure to visible light was discovered to contribute most to the photocatalytic effectiveness of textile fabrics modified with TiO_2_ and ZnO. It was demonstrated that natural lighting with intensity of 92.3–95.0 lux and lighting duration of 420 min/day contributes more to the reduction in the number of microorganisms in the air than artificial light with light intensity of 152.8–166.7 lux and lighting duration of 80.7–87.8 min/day. Similarly, Chen et al. [59] found that low light intensity with long exposure time was more effective in inactivating microbes than high light intensity with short exposure time with the same UV dose, which was defined as the product of intensity and irradiation time. Model studies carried out by other researchers revealed that the light exposure duration necessary for the inactivation of microorganisms in the air in different photocatalyst systems and at different light exposure times ranges from 15 min to 72 h [7,32]. The duration of exposure to visible light seems to be the key parameter determining the effectiveness of air cleaning of microorganisms. For the bathroom with natural lighting, this was 5.5 h longer than in the bathroom with artificial lighting. Moreover, the studies were carried out in winter, when natural lighting in the bathroom lasted for only 7 h/day. Most probably, photocatalytic activity in other seasons can become more intensive as a result of the extended duration of exposure to visible light.

The present studies revealed that the photocatalytic activity of modified towels supports the cleaning of air from microorganisms with a light intensity range between 92 and 167 lux. At these parameters, no relationship had higher effectiveness at higher intensity of visible light. One should bear in mind that the process of photocatalytic air cleaning under real conditions simultaneously affects a number of parameters, such as the kind of photocatalytic material, its surface, lighting intensity, type and time of light exposure, and air humidity [31]. Other parameters which can affect photocatalytic activity under real conditions include air pollution with dust, especially with organic compounds, air flow, types of building materials, structures and equipment in the rooms, and hygienic conditions of the room.

Studies carried out within this project reveal the significant impact of air humidity on photocatalytic processes. RH = 30–75% is considered to be optimum for effective photocatalysis, and a humidity increase in the presented range contributes to a rise in the photocatalytic inactivation of microorganisms [3,32]. Some papers describe high activity at RH = 45% [7], while others demonstrate that effectiveness is greatly reduced over RH = 75–85% [3,32]. In the present studies, high air humidity (RH = 65.6–66.8%) was observed in the bathroom with artificial lighting, where a reduction in the number of microorganisms was discovered. A slightly higher reduction in the number of microorganisms was observed in the air in the bathroom with natural lighting, where RH = 36–44.5%. The temperature in the tested bathrooms was 21–23 °C, which according to the literature, is not significant for photocatalysis [3].

In the present studies, the real conditions in the tested bathrooms, where the photocatalytic processes were conducted, were demonstrated as sufficient for reducing the number of microorganisms in the air by 1 log on average. The reduction in air microbiological contamination from 10^3^ to 10^2^ CFU/m^3^ for bacteria and from 10^2^ to 10^1^ CFU/m^3^ for fungi is a satisfactory result for the environment. Air was considered as non-contaminated under household conditions, which was the desired effect. Many microorganisms proliferate in air, including pathogenic bacteria, and toxinogenic and allergenic mold [60]. Removing bacteria and mold from the air in model photocatalytic systems in visible light has been the subject of a number of papers, which have demonstrated high antimicrobial effectiveness [53]. The present study covered textile fabrics modified with TiO_2_ and ZnO, used in bathrooms. The results suggest that the modification can support air cleaning. One should note that in the tested bathrooms with a volume of 12.86–40.5 m^3^ only two towels (size: 70 × 150 cm^2^) were used, which can affect photocatalytic effectiveness.

A reduction in gas concentration (NO_2_, CO_2_, CO) in the air in bathrooms where modified towels were used is testimony to the occurrence of photocatalytic processes. The number of VOCs and their concentration were also observed to decrease significantly above the modified towels compared to the control towels. The main VOCs were of cosmetic origin; however, some compounds of textile and microbiological origin were also observed. The literature provides evidence for the oxidation of such inorganic and organic compounds as dyes (methylene blue, rhodamine B, methyl orange, sunset yellow, Reactive Black 5 and Acid Orange 7), toxic compounds (cyanide, bisphenol A, 2,4 dichlorophenol, phenol, toluene, benzene and humic acid) and inorganic compounds (nitrate, bicarbonate, sulphate and NOx) under model conditions [16]. The oxidation of organic compounds was confirmed under real conditions when photocatalysis was used for the treatment of wastewater or river water and air [16]. This study is the first to be carried out under real conditions where a reduction in the concentration of inorganic and organic compounds in the air was demonstrated as a result of the activity of photocatalytically modified textile fabrics. Visible light was confirmed to be able to induce photocatalysis, which effectively removes some VOCs and undesired gases from the air. Moreover, observations of the modified towels during seven days of use revealed lack of odors, compared to the control towels.

Due to the type and application of the tested terry towels, they should be relatively often washed at high temperatures. Hence, the modification should be resistant to washing. The results of washing fastness studies will be the subject of a next study.

## 5. Conclusions

The results of the study suggest that textile fabrics modified with TiO_2_ and ZnO nanoparticles can be used as self-cleaning towels. The low and medium antimicrobial effectiveness of photocatalytically modified TiO_2_ and ZnO textile materials under model conditions was revealed, alongside with high effectiveness under real conditions. Significant biostatic activity was shown on the surface of the modified towels, where the number of microorganisms decreased by 1–5 log to a level of 0–5 CFU/cm^2^ for the following bacteria: *Enterobacteriaceae*, *Enterococcus*, coli group and *E. coli*, and *Pseudomonas*. Furthermore, a statistically significant decrease was found for the total number of fungi and bacteria.

Moreover, fabrics in visible lighting conditions can support air cleaning by reducing the number of bacteria and fungi, and the concentration of gases and VOCs in the air. A statistically significant reduction in the total number of bacteria and fungi (by 1 log) and the concentration of gases (NO_2_, CO_2_, CO) in the air of bathrooms where modified towels were used, was determined. The removal of VOCs or a reduction in their concentration in the air above the modified towels was determined. Under real conditions, in the humidity range RH = 36–67% and light intensity between 81 and 167 lux, the type and time of lighting were observed to be more significant for the effectiveness of photocatalysis than air humidity. Greater photocatalytic effectiveness was revealed in natural light during 7 h/day than in artificial light during 1.5 h/day.

Future studies should focus on determining the durability of the photocatalytic effects of modified textile fabrics after washing. Furthermore, studies should be continued to optimize microclimatic conditions and lighting with visible light in order to improve the photocatalytic effects.

## Figures and Tables

**Table 1:** Size and particle size distribution of ZnO and TiO_2_ NPs in commercial dispersions as obtained by the DLS analysis.

NPs	Fraction	Volume Weighted		Number Weighted	
		Diameter (nm)	Percentage (%)	Diameter (nm)	Percentage (%)
ZnO	1	7.1	87.2	7.0	96.0
	2	23.8	12.7	23.0	4.0
	3	181.0	0.1	168.2	0.0
TiO_2_	1	6.3	85.4	6.3	95.3
	2	21.4	14.6	20.8	4.7

**Table 2:** Selected properties and color fastness of terry cotton fabrics.

Textiles	Surface Mass (g/m^2^)	Time of Absorption (s)	Color Fastness			
			Washing in 60 °C		Rubbing	
			Color Change	Staining	Dry	Wet
control	518	5.21	5	5	5	5
modified	540	5.08	5	5	5	5

**Table 3:** Self-cleaning properties.

Textiles	K/S Values (540 nm)		ΔE*	L*		a*		b*	
	BI	A6I		BI	A6I	BI	A6I	BI	A6I
control	0.50	0.37	6.145	73.38	75.44	15.23	10.35	−2.22	0.88
modified	0.42	0.17	12.905	73.57	80.70	9.56	2.52	−1.86	6.28

BI—before irradiation, A6I—after 6 h of irradiation.

**Table 4:** Characteristics of microclimatic and lighting conditions in bathrooms during seven days of towel use—real conditions.

Day	Bathroom with Artificial Lighting No. 1				Bathroom with Natural Lighting No. 2			
	RH (%)	Temp (°C)	Light Intensity (lux)	Lighting Time (min/24 h)	RH (%)	Temp (°C)	Light Intensity (lux)	Lighting Time (min/24 h)
**Control Towels**								
1	58.8	22.0	173.0	55	46.0	22.3	61.9	420
2	67.2	21.1	165.6	70	43.4	23.7	62.5	420
3	62.1	21.2	156.3	100	40.6	23.8	73.5	420
4	63.8	20.8	160.3	105	44.7	23.7	70.1	420
5	62.7	20.3	171.3	110	50.4	20.3	119.3	420
6	68.7	22.5	169.7	100	42.3	23.7	69.2	420
7	78.7	21.9	171.0	75	43.8	23.7	208.3	420
M:	65.6	21.4	166.7	87.86	44.5	23.0	95.0	420
**Modified Towels**								
1	66.5	21.8	156.6	50	39.6	24.1	64.4	420
2	68.5	21.3	165.0	90	39.8	23.6	96.6	420
3	65.0	21.4	163.0	75	39.6	24.1	90.9	420
4	64.6	20.6	152.0	100	32.5	23.9	88.0	420
5	64.4	21.4	145.0	75	32.0	23.5	103.4	420
6	65.1	20.7	143.0	85	34.8	23.1	99.6	420
7	73.8	23.4	145.0	90	33.7	23.4	103.0	420
M:	66.8	21.5	152.8	80.7	36.0	23.7	92.3	420

M—mean; min—minutes, temp—temperature.

**Table 5:** Microbial contamination of air and gas concentration in the air during seven days of towel use in different light conditions.

Towels/Light Conditions	Days	Number of Microorganisms in the Air During Use of Towels		Gas Concentrations in the Air During Use of Towels		
		Bacteria (CFU/m^3^)	Fungi (CFU/m^3^)	NO_2_ (ppm)	CO_2_ (vol%)	CO (ppm)
Control/artificial light	1	M: 2.19 × 10^3^ SD: 3.65 × 10^2^	M: 1.77 × 10^2^ SD: 5.51 × 10^1^	M: 0.46 SD: 0.06	M: 0.14 SD: 0.01	M: 33.67 SD: 4.51
	4	M: 1.84 × 10^3^ SD: 3.28 × 10^2^	M: 1.21 × 10^2^ SD: 1.79 × 10^1^	M: 0.43 SD: 0.11	M: 0.18 SD: 0.01	M: 32.33 SD: 4.16
	7	M: 2.13 × 10^3^ SD: 2.80 × 10^2^	M: 1.37 × 10^2^ SD: 1.55 × 10^1^	M: 0.43 SD: 0.12	M: 0.17 SD: 0.01	M: 40.33 SD: 5.86
	M;SD	**M: 2.05 × 10^3 #^ SD: 1.87 × 10^2^**	**M: 1.45 × 10^2 #^ SD: 1.55 × 10^1^**	**M: 0.44 SD: 0.02**	**M: 0.16 SD: 0.02**	**M: 35.44 SD: 4.28**
Control/natural light	1	M: 1.69 × 10^3^ SD: 3.91 × 10^2^	M: 4.67 × 10^1^ SD: 1.04 × 10^1^	M: 0.02 SD: 0.00	M: 0.12 SD: 0.00	M: 25.33 SD:12.58
	4	M: 1.17 × 10^3^ SD: 1.94 × 10^2^	M: 9.67 × 10^1^ SD: 1.03 × 10^1^	M: 0.03 SD: 0.01	M: 0.13 SD: 0.01	M: 20.33 SD: 4.04
	7	M: 1.55 × 10^3^ SD: 4.59 × 10^2^	M: 4.87 × 10^1^ SD: 1.63 × 10^1^	M: 0.02 SD: 0.01	M: 0.11 SD: 0.01	M: 21.00 SD: 1.00
	M;SD	**M: 1.47 × 10^3 #^ SD: 2.69 × 10^2^**	**M: 6.40 × 10^1^ SD: 2.83 × 10^1^**	**M: 0.02 SD: 0.01**	**M: 0.12 SD: 0.01**	**M: 22.22 SD: 2.71**
Modified/artificial light	1	M: 1.12 × 10^3^ SD: 1.23 × 10^2^	M: 7.00 × 10^1^ SD: 2.65 × 10^1^	M: 0.33 SD: 0.06	M: 0.13 SD: 0.01	M: 43.00 SD: 7.00
	4	M: 8.33 × 10^2^ SD: 2.52 × 10^2^	M: 2.33 × 10^1^ SD: 5.77 × 10^1^	M: 0.00 SD: 0.00	M: 0.11 SD: 0.01	M: 10.67 SD: 1.53
	7	M: 1.39 × 10^2^ SD: 1.67 × 10^1^	M: 1.33 × 10^1^ SD: 5.72 × 10^0^	M: 0.00 SD: 0.00	M: 0.13 SD: 0.02	M: 16.66 SD: 1.52
	M;SD	M: 6.97 × 10^2,^* SD: 5.04 × 10^2^	M: 3.55 × 10^1,^* SD: 3.03 × 10^1^	M: 0.11 * SD: 0.19	M: 0.12 * SD: 0.01	M: 23.44 * SD: 7.20
Modified/natural light	1	M: 2.92 × 10^2^ SD: 7.65 × 10^1^	M: 3.00 × 10^1^ SD: 4.00 × 10^0^	M: 0.00 SD: 0.00	M: 0.11 SD: 0.01	M: 10.33 SD: 1.53
	4	M: 3.25 × 10^2^ SD: 4.27 × 10^1^	M: 5.33 × 10^0^ SD: 2.31 × 10^0^	M: 0.00 SD: 0.00	M: 0.08 SD: 0.00	M: 6.66 SD: 1.15
	7	M: 4.20 × 10^2^ SD: 2.78 × 10^1^	M: 6.67 × 10^0^ SD: 3.06 × 10^0^	M: 0.00 SD: 0.00	M: 0.08 SD: 0.01	M: 8.00 SD: 1.00
	M;SD	M: 3.46 × 10^2,^* SD: 6.65 × 10^1^	M: 1.40 × 10^1,^* SD: 1.39 × 10^1^	M: 0.00 * SD: 0.00	M: 0.09 * SD: 0.01	M: 8.33 * SD: 1.86

M—mean, SD—standard deviation; **^#^** high number of bacteria > 10^3^ CFU/m^3^; high number of fungi > 10^2^ CFU/m^3^; * significant reduction of microorganism number in air/gas concentration in air during use of the modified towel compared to the control towel.

**Table 6:** Volatile organic compounds identified in the air above towels (control and modified) after seven days of use in different light conditions.

No.	Volatile Organic Compounds (VOCs)	Control		Modified		Area Reduction in Modified Sample/VOCs Origin *
		Area	% of Total Area	Area	% of Total Area	
1	Ethanol	64,209	0.37	623,788	4.42	−/M, C
2	Isopropanol	329,104	1.24	58,626	0.43	+/M, C
3	2-Butanone	25,785	0.10	87,122	0.64	−/M, C
4	Trichloromethane (syn. Chloroform)	131,788	0.76	35,410	0.25	+/T
5	1-Butanol	nd	nd	134,561	0.99	−/M, C, T
6	3-Methyl-1-butanol	18,525	0.11	43,211	0.31	−/M, C
7	2-Methyl-1-butanol	6066	0.04	14,627	0.10	−/M, C, T
8	1-Pentanol	12,684	0.09	12,400	0.09	+/−/M, C, T
9	Toluene	85,973	0.32	107,123	0.78	−/M, C, T
10	Hexanal	165,412	0.63	133,358	0.98	−/M, C, T
11	Acetic acid, butyl ester	19,278	0.11	15,233	0.11	+/−/M, C
12	1-hexanol	57,097	0.33	5508	0.04	+/M, C
13	Butanoic acid, ethyl ester	18,687	0.07	2058	0.07	+/−/M, C
14	2-Pentanone, 4-hydroxy-4-methyl- (syn. Diacetone/Acetonyldimethylcarbinol)	nd	nd	16,505	0.12	−/C
15	2-Heptanone	34,209	0.13	5301	0.04	+/M
16	Heptanal	31,581	0.18	17,058	0.12	+/M
17	Benzaldehyde	38,333	0.23	31,922	0.23	+/−/M, C, T
18	2-Ethylcaproaldehyde	14,812	0.06	9650	0.06	+/−/C
19	2-Ethyltoluene	14,720	0.09	8750	0.06	+/T
20	1-Heptanol	30,417	0.18	5704	0.04	+/C, T
21	6-Methyl-5-heptene-2-one	58,622	0.22	36,606	0.27	−/C
22	2-Octanone	66,411	0.39	nd	nd	+/M, C, T
23	Octanal	60,464	0.35	65,850	0.47	−/M, C, T
24	Hexanoic acid, ethyl ester (syn. Ethyl caproate)	31,829	0.18	7939	0.06	+/M, C
25	1-(2-Methoxypropoxy)-2-propanol	66,640	0.39	7530	0.05	+/C
26	Benzyl alcohol	16,420.08	0.06	22,993	0.17	−/M, C, T
27	Nitrohexane	24,880	0.09	nd	nd	+/T
28	1-Hexanol, 2-ethyl-	4,338,990	16.40	971,096	7.11	+/C
29	Fenchene	7860	0.03	nd	nd	+/C
30	2-Ethylhexanol	1,952,921	11.32	nd	nd	+/M, C, T
31	3-methyl-6-hepten-1-ol	3413	0.02	nd	nd	+/M, T
32	1-Octanol	238,954	1.39	54,462	0.39	+/M, C, T
33	7-Octen-2-ol, 2,6-dimethyl- (syn. Dihydromyrcenol)	1,460,458	5.52	607,055	4.45	+/C, T
34	Nonanal	472,936	1.79	580,980	4.25	−/M, C, T
35	1,6-Octadien-3-ol, 3,7-dimethyl-, formate	2,164,449	8.18	567,510	4.16	+/C
36	3-Octanol, 3,7-dimethyl- (syn. Tetrahydrolinalool)	268,812	1.56	813,728	5.77	−/C, T
37	Fenchol	13,311	0.05	nd	nd	+/C
38	Methyl caprylate	7905.46	0.03	nd	nd	+/M, C
39	Acetic acid, phenylmethyl ester	91,453	0.35	99,061	0.73	−/M, C
40	Ethylene glycol monohexyl ether	14,127	0.08	10,749	0.08	+/−/C
41	Benzene, 1-ethenyl-4-methoxy- (syn. 4-Vinylanisole)	158,174	0.92	22,008	0.16	+/C
42	D-isomenthone	62,544	0.36	26,520	0.19	+/C
43	5-Caranol, trans,trans-(+)-	33,303	0.19	9872	0.07	+/C
44	Benzoic acid, ethyl ester	406,315	2.36	54,968	0.39	+/C, T
45	Ethanol, 1-(2-butoxyethoxy)-	189,056	0.71	2,195,398	16.08	−/C
46	Benzenemethanol, .alpha.-methyl-, acetate (syn. Gardenol)	93,553	0.35	161,549	1.18	−/C
47	Cyclohexanol, 5-methyl-2-(1-methylethyl)- (syn. Menthol)	3,332,650	19.32	1,016,358	7.21	+/C
48	Ethanol, 2-(2-butoxyethoxy)- (syn. 1-(2-Butoxyethoxy)ethanol)	–	–	177,022	1.26	−/C
49	1,6-Nonadien-3-ol, 3,7-dimethyl- (syn. Ethyl linalool)	23,904	0.14	32,311	0.23	−/C
50	Octanoic acid, ethyl ester	477,556	1.80	231,696	1.80	+/−/C
51	Decanal	194,542	1.13	314,444	2.23	−/M, C, T
52	Benzisothiazole	92,810	0.35	30,850	0.23	+/T
53	Geranyl vinyl ether	36,582	0.14	17,726	0.14	+/−/C
54	Cyclohexanol, 4-(1,1-dimethylethyl)-, trans-	23,053	0.10	13,632	0.10	+/−/T
55	p-tert-Butylcyclohexanol	38,808	0.23	nd	nd	+/T
56	Citronellol	1,546,034	5.84	530,238	3.88	+/C
57	D-Carvone	140,583	0.53	50,096	0.37	+/C
58	Octanoic acid, octyl ester	49,441	0.19	nd	nd	+/C
59	Geraniol	529,339	2.00	36,022	0.26	+/C
60	Linalyl acetate	52,116	0.30	28,266	0.20	+/C
61	Citral	16,907	0.06	2547	0.02	+/C
62	1-Decanol	446,504	2.59	134,082	0.95	+/M, C, T
63	Anethole	92,568	0.54	12,841	0.09	+/C
64	Naphthalene, 1-methyl -	313,028	1.81	207,695	1.81	+/−/T
65	2-Undecanone (syn. Methyl nonyl ketone)	34,336	0.13	nd	nd	+/C
66	Naphthalene, 2-methyl-	162,470	0.94	119,947	0.85	+/T
67	Benzeneethanol, .alpha.,.alpha.-dimethyl-, acetate (syn. Dimethylbenzylcarbinol acetate)	41,065	0.24	12,481	0.09	+/c
68	α-Terpineol acetate	114,508	0.43	45,361	0.33	+/C
69	2,2-Dimethyl-1-(2-hydroxy-1-isopropyl)propyl ester of isobutanoic acid	310,758	1.80	126,974	0.90	+/C
70	3-Hydroxy-2,2,4-trimethylpentyl ester of isobutanoic acid	360,979	2.09	176,147	1.25	+/C
71	Lavandulol acetate	21,712	0.09	12,519	0.09	+/−/C
72	Decanoic acid, ethyl ester	176,791	1.03	33,981	0.24	+/C
73	Dodecanal	86,895	0.50	57,221	0.41	+/M, C
74	Decyl isobutyl carbonate	691,817	2.61	nd	nd	+/C
75	1-dodecanol	1,542,500	5.83	1,375,030	10.07	−/C
76	Nerylacetone	198,334	1.15	130,671	0.93	+/C
77	1-Tetradecanol	1,751,286	10.15	934,744	6.63	+/C
78	Butanoic acid, 1,1-dimethyl-2-phenylethyl ester (syn. Benzyl dimethylcarbinyl butyrate)	70,933	0.41	33,589	0.24	+/C
79	Benzenepentanoic acid, .beta.-methyl-, ethyl ester, (S)-	52,736	0.31	66,828	0.47	−/C
80	dibenzo-furan	187,609	1.09	184,077	1.31	−/C
81	Indan-1,3-diol monopropionate	114,311	0.66	nd	nd	+/C
82	Lily aldehyde	2,550,199	9.64	862,292	6.31	+/C
83	Alpha-N-Methyl Ionone	49,703	0.29	49,168	0.35	−/C
84	Dodecanoic acid, methyl ester	81,482	0.31	7575	0.06	+/T
85	2(3H)-Furanone, 5-heptyldihydro- (syn. γ-Heptylbutyrolactone)	41,414	0.16	17,536	0.16	+/−/C
86	γ-Dodecalactone	37,804	0.22	15,687	0.11	+/C
87	Diethyl Phthalate	89,114	0.34	66,482	0.49	−/C, T
88	Methyl P-T- Butylphenylacetate	30,393	0.18	nd	nd	+/C
89	Dodecanoic acid, ethyl ester	183,265	1.06	36,601	0.26	+/T
90	Propanoic acid, 2-methyl-, 1-(1,1-dimethylethyl)-2-methyl-1,3-propanediyl ester	120,403	0.45	285,712	2.09	−/T
91	Methanone, diphenyl- (syn. Benzophenone)	30,428	0.11	57,138	0.42	−/C
92	Cedrol	50,713	0.19	73,951	0.54	−/C
93	Hexadecane	74,426	0.28	50,004	0.37	−/C
94	Dodecanoic acid, 1-methylethyl ester (syn. Isopropyl laurate)	24,175	0.09	18,273	0.13	−/C, T
95	Heptadecane	218,568	0.83	58,643	0.43	+/C
96	2-methyl-propanoic acid, 2,2-dimethyl-1-(1-methylethyl)-1,3-propanediyl ester	376,511	2.18	277,672	1.97	+/C
97	Octanal, 2-(phenylmethylene)- (syn. α-Hexylcinnamaldehyde)	660,220	3.83	466,424	3.81	+/−/C
98	Benzoic acid, phenylmethyl ester	175,155	0.66	132,425	0.97	−/C
99	Heptadecanoic acid, ethyl ester	14,993	0.06	15,798	0.12	−/M
100	Tetradecanoic acid, ethyl ester	73,941	0.43	11,691	0.08	+/T
101	Isopropyl myristate	246,431	1.43	119,968	0.85	+/C, T
102	Tonalid	38,855	0.23	27,988	0.23	+/C
103	Phthalic acid, hept-4-yl isobutyl ester	2,482,769	9.38	131,723	0.96	+/C
104	Hexadecanoic acid, methyl ester	1,038,878	6.02	20,464	0.15	+/T
105	Phthalic acid, butyl hex-3-yl ester	763,942	2.89	64,088	0.47	+/C
106	Hexanedioic acid, bis(2-ethylhexyl) ester	251,496	1.46	nd	nd	+/C
107	Hexadecanoic acid, ethyl ester	75,788	0.44	12,541	0.09	+/T
108	Ethylene brassylate	112,516	0.65	105,067	0.75	−/C, T
109	Isopropyl palmitate	59,876	0.35	nd	nd	+/C, T
110	Dodecanoic acid, isooctyl ester	58,760	0.22	nd	nd	+/C
111	Methyl stearate	46,919	0.27	nd	nd	+/C
	Summary:					+66 −32 +/−13

* VOCs origin: M—microbial, C—cosmetic, T—textile on the basis of the literature [1,2,3,4,5,6,7,8,9,10,11,12,13,14,15,16,17,18,19,20,21,22,23,24,25]; VOCs identified in all samples (four replicates) marked in grey color; VOCs identified in control (without use of towels) and modified samples (origin T—textiles) marked in cream color; (+) the area under peak (VOCs concentration) decreased in modified sample compared to the control sample; (−) the area under peak (VOCs concentration) increased in modified sample compared to the control sample; (+/−) the areas under peak (VOCs concentration) in the modified and control samples are the same; nd—not detected.

## References

[1] Forman H.J., Augusto O., Brigelius-Flohé R., Dennery P.A., Kalyanaraman B., Ischiropoulos H., Mann G., Radi R., Roberts L.J., Viña J. (2015). Even free radicals should follow some rules: A Guide to free radical research terminology and methodology. Free. Radic. Boil. Med..

[2] Wang J., Zhao J., Sun L., Wang X. (2014). A review on the application of photocatalytic materials on textiles. Text. Res. J..

[3] Goswami D.Y., Trivedi D.M., Block S.S. (1997). Photocatalytic Disinfection of Indoor Air. J. Sol. Energy Eng..

[4] Mccullagh C., Robertson J.M.C., Bahnemann D.W., Robertson P.K.J. (2007). The application of TiO_2_ photocatalysis for disinfection of water contaminated with pathogenic micro-organisms: A review. Res. Chem. Intermed..

[5] Gamage J., Meng X. (2010). Applications of Photocatalytic Disinfection. Int. J. Photoenergy.

[6] Foster H.A., Ditta I.B., Varghese S., Steele A. (2011). Photocatalytic disinfection using titanium dioxide: Spectrum and mechanism of antimicrobial activity. Appl. Microbiol. Biotechnol..

[7] Muranyi P., Schraml C., Wunderlich J. (2009). Antimicrobial efficiency of titanium dioxide-coated surfaces. J. Appl. Microbiol..

[8] Radetić M. (2013). Functionalization of textile materials with TiO_2_ nanoparticles. J. Photochem. Photobiol. C: Photochem. Rev..

[9] Yuranova T., Mosteo R., Bandura J., Laub D., Kiwi J. (2006). Self-cleaning cotton textiles surfaces modified by photoactive SiO_2_/TiO_2_ coating. J. Mol. Catal. A: Chem..

[10] Landi S., Carneiro J., Ferdov S., Fonseca A.M., Neves I.C., Ferreira M., Parpot P., Soares O.S.G.P., Pereira M.F.R. (2017). Photocatalytic degradation of Rodamine B dye by cotton textile coated with SiO_2_-TiO_2_ and SiO_2_-TiO_2_-HY composites. J. Photochem..

[11] Wang R., Wang X., Xin J.H. (2009). Advanced Visible-Light-Driven Self-Cleaning Cotton by Au/TiO_2_/SiO_2_ Photocatalysts. ACS Appl. Mater. Interfaces.

[12] Behzadnia A., Montazer M., Rad M.M. (2015). Simultaneous sonosynthesis and sonofabrication of N-doped ZnO/TiO_2_ core–shell nanocomposite on wool fabric: Introducing various properties specially nano photo bleaching. Ultrason. Sonochem..

[13] Mihailovic D., Saponjic Z., Radoicic M., Molina R., Radetić T., Jovančić P., Nedeljković J.M., Radetić M. (2011). Novel properties of PES fabrics modified by corona discharge and colloidal TiO_2_ nanoparticles. Polym. Adv. Technol..

[14] Yuranova T., Laub D., Kiwi J. (2007). Synthesis, activity and characterization of textiles showing self-cleaning activity under daylight irradiation. Catal. Today.

[15] Pablos C., Marugán J., Van Grieken R., Dunlop P., Hamilton J., Dionysiou D.D., Byrne J.A. (2017). Electrochemical Enhancement of Photocatalytic Disinfection on Aligned TiO_2_ and Nitrogen Doped TiO_2_ Nanotubes. Molecules.

[16] Demirel C.S.U., Birben N., Bekbolet M. (2018). A comprehensive review on the use of second generation TiO_2_ photocatalysts: Microorganism inactivation. Chemosphere.

[17] Verbič A., Gorjanc M., Simončič B. (2019). Zinc Oxide for Functional Textile Coatings: Recent Advances. Coatings.

[18] Zhang X., Qin J., Hao R., Wang L.-M., Shen X., Yu R., Limpanart S., Ma M., Liu R. (2015). Carbon-Doped ZnO Nanostructures: Facile Synthesis and Visible Light Photocatalytic Applications. J. Phys. Chem. C.

[19] Krishna M.G., Vinjanampati M., Purkayastha D.D. (2013). Metal oxide thin films and nanostructures for self-cleaning applications: Current status and future prospects. Eur. Phys. J. Appl. Phys..

[20] Li X., Wang C., Xia N., Jiang M., Liu R., Huang J., Li Q., Luo Z., Liu L., Xu W. (2017). Novel ZnO-TiO_2_ nanocomposite arrays on Ti fabric for enhanced photocatalytic application. J. Mol. Struct..

[21] Li D., Jiang X., Zhangb Y., Zhang B., Pan C. (2012). A novel route to ZnO/TiO_2_ heterojunction composite fibers. J. Mater. Res..

[22] Carré G., Benhamida D., Peluso J., Muller C.D., Lett M.-C., Gies J.-P., Keller V., Keller N., Andre P. (2013). On the use of capillary cytometry for assessing the bactericidal effect of TiO_2_. Identification and involvement of reactive oxygen species. Photochem. Photobiol. Sci..

[23] Jones N., Ray B., Ranjit K.T., Manna A.C. (2008). Antibacterial activity of ZnO nanoparticle suspensions on a broad spectrum of microorganisms. FEMS Microbiol. Lett..

[24] Carré G., Hamon E., Ennahar S., Estner M., Lett M.-C., Horvatovich P., Gies J.-P., Keller V., Keller N., Andre P. (2014). TiO_2_ Photocatalysis Damages Lipids and Proteins in Escherichia coli. Appl. Environ. Microbiol..

[25] Chu K.H., Huang G., An T., Li G., Yip P.L., Ng T.W., Yip H.Y., Zhao H., Wong P.K. (2016). Photocatalytic inactivation of Escherichia coli -the roles of genes in boxidation of fatty acid degradation. Catal. Today.

[26] Luttrell T., Halpegamage S., Tao J., Kramer A., Sutter E., Batzill M. (2014). Why is anatase a better photocatalyst than rutile?—Model studies on epitaxial TiO_2_ films. Sci. Rep..

[27] Acayanka E., Tarkwa J.-B., Nchimi K.N., Voufouo S.A., Tiya-Djowe A., Kamgang G.Y., Laminsi S. (2019). Grafting of N-doped titania nanoparticles synthesized by the plasma-assisted method on textile surface for sunlight photocatalytic self-cleaning applications. Surf. Interfaces.

[28] Shaban M., Mohamed F., Abdallah S. (2018). Production and Characterization of Superhydrophobic and Antibacterial Coated Fabrics Utilizing ZnO Nanocatalyst. Sci. Rep..

[29] Ko G., First M., Burge H. (2000). Influence of relative humidity on particle size and UV sensitivity of Serratia marcescens and Mycobacterium bovis BCG aerosols. Tuber. Lung Dis..

[30] Gogniat G., Thyssen M., Denis M., Pulgarin C., Dukan S. (2006). The bactericidal effect of TiO_2_ photocatalysis involves adsorption onto catalyst and the loss of membrane integrity. FEMS Microbiol. Lett..

[31] Chen F., Yang X., Mak H.K., Chan D.W. (2010). Photocatalytic oxidation for antimicrobial control in built environment: A brief literature overview. Build. Environ..

[32] Bogdan J., Zarzyńska J., Pławińska-Czarnak J. (2015). Comparison of Infectious Agents Susceptibility to Photocatalytic Effects of Nanosized Titanium and Zinc Oxides: A Practical Approach. Nanoscale Res. Lett..

[33] Vohra A., Goswami D.Y., Deshpande D.A., Block S.S. (2005). Enhanced photocatalytic inactivation of bacterial spores on surfaces in air. J. Ind. Microbiol. Biotechnol..

[34] Qian T., Su H., Tan T. (2011). The bactericidal and mildew-proof activity of a TiO_2_–chitosan composite. J. Photochem. Photobiol. A: Chem..

[35] Znaidi L. (2010). Sol–gel-deposited ZnO thin films: A review. Mater. Sci. Eng. B.

[36] ISO (2000). PN-EN 12127:2000, Textiles—Fabrics—Determination of Mass per Unit Area Using Small Samples.

[37] ISO (2007). Annex B: Determination of time of absorption. PN-EN 14697:2007 Textile—Terry Towels and Terry Towel Fabrics—Specification and Methods of Testing.

[38] ISO (2010). PN-EN ISO 105-C06:2010, Washing Fastness to Dry and Wet Rubbing.

[39] ISO (2016). PN-EN ISO 105-X12:2016, Textiles—Tests for Colour Fastness—Part X12: Colour Fastness to Rubbing.

[40] AATCC (2010). AATCC Test Method 100-2004, Antimicrobial Finishes on Textile Materials: Assessment of Antimicrobial Finishes on Textile Materials, Technical Manual/2010.2004.

[41] Majchrzycka K., Okrasa M., Skora J., Gutarowska B. (2016). Evaluation of the Survivability of Microorganisms Deposited on Filtering Respiratory Protective Devices under Varying Conditions of Humidity. Int. J. Environ. Res. Public Health.

[42] ISO (2019). PN-EN 1276:2019-12, Chemical Disinfectants and Antiseptics—Quantitative Suspension Method for Determining the Bactericidal Effect of Chemical Disinfectants and Antiseptics Used in the Food Sector, Industrial and Domestic Conditions and Public Utilities—Test Method and Requirements.

[43] He J., Du Y.-E., Bai Y., An J., Cai X., Chen Y., Wang P., Yang X., Feng Q. (2019). Facile Formation of Anatase/Rutile TiO_2_ Nanocomposites with Enhanced Photocatalytic Activity. Molecules.

[44] Ijadpanah-Saravy H., Safari M., Khodadadi-Darban A., Rezaei A. (2014). Synthesis of Titanium Dioxide Nanoparticles for Photocatalytic Degradation of Cyanide in Wastewater. Anal. Lett..

[45] Bacciarelli-Ulacha A., Rybicki E., Matyjas-Zgondek E., Pawlaczyk A., Szynkowska M.I. (2014). A New Method of Finishing of Cotton Fabric by in Situ Synthesis of Silver Nanoparticles. Ind. Eng. Chem. Res..

[46] Zhang L., Dillert R., Bahnemann D., Vormoor M. (2012). Photo-induced hydrophilicity and self-cleaning: Models and reality. Energy Environ. Sci..

[47] Pal A., Pehkonen S., Yu L., Ray M.B. (2007). Photocatalytic inactivation of Gram-positive and Gram-negative bacteria using fluorescent light. J. Photochem. Photobiol. A Chem..

[48] Seven O., Dindar B., Aydemir S., Metin D., Ozinel M., Icli S. (2004). Solar photocatalytic disinfection of a group of bacteria and fungi aqueous suspensions with TiO_2_, ZnO and Sahara desert dust. J. Photochem. Photobiol. A Chem..

[49] Markowska-Szczupak A., Ulfig K., Morawski A.W. (2011). The application of titanium dioxide for deactivation of bioparticulates: An overview. Catal. Today.

[50] Zhao J., Yang X. (2003). Photocatalytic oxidation for indoor air purification: A literature review. Build. Environ..

[51] Bonetta S., Bonetta S., Motta F., Strini A., Carraro E. (2013). Photocatalytic bacterial inactivation by TiO_2_-coated surfaces. AMB Express.

[52] Bazant P., Sedlacek T., Kuritka I., Podlipny D., Holcapkova P. (2018). Synthesis and Effect of Hierarchically Structured Ag-ZnO Hybrid on the Surface Antibacterial Activity of a Propylene-Based Elastomer Blends. Materials.

[53] Wysocka I., Markowska-Szczupak A., Szweda P., Ryl J., Endo-Kimura M., Kowalska E., Nowaczyk G., Zielińska-Jurek A. (2019). Gas-phase removal of indoor volatile organic compounds and airborne microorganisms over mono- and bimetal-modified (Pt, Cu, Ag) titanium(IV) oxide nanocomposites. Indoor Air.

[54] Karami A., Xie Z., Zhang J., Kabir M.S., Munroe P., Kidd S.P., Zhang H. (2020). Insights into the antimicrobial mechanism of Ag and I incorporated ZnO nanoparticle derivatives under visible light. Mater. Sci. Eng. C.

[55] Yang L., Liu Z. (2007). Study on light intensity in the process of photocatalytic degradation of indoor gaseous formaldehyde for saving energy. Energy Convers. Manag..

[56] Sunada K., Kikuchi Y., Hashimoto K., Fujishima A. (1998). Bactericidal and Detoxification Effects of TiO_2_ Thin Film Photocatalysts. Environ. Sci. Technol..

[57] Huang Z., Maness P.-C., Blake D.M., Wolfrum E.J., Smolinski S.L., Jacoby W.A. (2000). Bactericidal mode of titanium dioxide photocatalysis. J. Photochem. Photobiol. A: Chem..

[58] Fujishima A., Zhang X. (2006). Titanium dioxide photocatalysis: Present situation and future approaches. Comptes Rendus Chim..

[59] Chen F.N., Yang X., Wu Q. (2009). Photocatalytic oxidation of Escherichia coli, Aspergillus niger and formaldehyde under different UV irradiation conditions. Environ. Sci. Technol..

[60] Lin C.-Y., Li C.-S. (2003). Inactivation of Microorganisms on the Photocatalytic Surfaces in Air. Aerosol Sci. Technol..

